# The Crystallization Mechanism of Zr-Based Bulk Metallic Glass during Electron Beam Remelting

**DOI:** 10.3390/ma13163488

**Published:** 2020-08-07

**Authors:** Xiaopeng Li, Zeyu Zhang, Yi Yang, Jikang Fan, Jian Kong, Kehong Wang

**Affiliations:** College of Material Science and Engineering, Nanjing University of Science and Technology, Nanjing 210094, China; lxp11s00914@163.com (Z.Z.); 13B909097@hit.edu.cn (Y.Y.); wangllnjust@126.com (J.K.); wkh1602@126.com (K.W.)

**Keywords:** electron beam remelting, BMGs, microstructures, critical cooling or heating rate, FEM

## Abstract

Bulk metallic glasses (BMGs) are promising for multifunctional and structural application in different industries. However, the limited size of BMGs hinders their further application. The welding of BMGs has shown the possibility of getting rid of the casting size limitation. The heat-affected zone (HAZ) and fusion zone (FZ) often undergo severe crystallization during the welding process. It is still unclear whether the crystallization occurs during the heating process or the cooling process. To figure out the crystallization mechanisms of Zr-based BMGs during the electron beam welding process, the Zr-based BMGs with the composition of Zr_41.2_Ti_13.8_Cu_12.5_Ni_10_Be_22.5_ were remelted by electron beam. The microstructures of the HAZ and the remelting zone (RZ) were analyzed. The thermal field of the electron beam welding was obtained by the finite element method (FEM). The critical conditions for crystallization during the heating and cooling processes were obtained by differential scanning calorimetry (DSC) and the Kissinger equation. The results show that the Zr_41.2_Ti_13.8_Cu_12.5_Ni_10_Be_22.5_ in the HAZ undergoes severe crystallization, while the Zr_41.2_Ti_13.8_Cu_12.5_Ni_10_Be_22.5_ in RZ keeps amorphous state after the remelting process. The low cooling rate in the HAZ is responsible for its crystallization.

## 1. Introduction

Bulk metallic glasses (BMGs) have won wide attention due to their better physical, chemical, and mechanical properties compared to normal crystal, which results from disordered atomic structures inherited from molten melts. Therefore, BMGs are promising for multifunctional and structural application in different industries, such as electrical products and sporting goods [1,2,3]. However, the limited size of BMGs hinders their further application [4]. Thus, it is necessary to find an effective way to break through the size restriction of BMGs.

To date, welding has proved to be an effective method to get large size BMGs. For instance, Yokoyama et al. achieved Zr_50_Cu_30_Ni_10_Al_10_ bulk glassy plates by using electron beam welding to suppress the grains in the joints [5]. Kawahito et al. also used a focused fiber laser beam to weld Zr-based BMGs, and the heat-affected zone (HAZ) remained desirably amorphous [6]. However, the BMGs often undergo severe crystallization during the welding process. The research of R.T. Mousavian et al. showed that some nano B_2_ precipitates formed in the HAZ of laser-remelted Zr_65.7_Ti_3.3_Al_3.7_Ni_11.7_Cu_15.6_ (wt.%) [7]. Sung Hwan Hong et al. discussed the reasons for the B_2_ precipitates [8]. Similarly, as Swiston et al. demonstrated, fast cooling is required in order to avoid crystallization, which is generally challenging since the local melting results in a larger heat-affected zone (HAZ) [9]. These studies mainly focused on the influence of welding parameters on the microstructures and mechanical properties of the joints, and lack of research on the mechanism of crystallization. It is still unclear whether the crystallization occurs during the heating process or the cooling process. Recently, Wang G. et al. [10] and Wang HS et al. [11] have studied the crystallization mechanisms of Ti-based bulk metallic glass in both the heating and cooling processes, which have great significance in the optimization of the welding process parameters. However, the crystallization kinetics and thermal stability of Zr-based BMGs have been rarely reported during the integral heating and cooling processes of welding to date [12,13,14].

To figure out the crystallization mechanisms of Zr-based BMGs, a typical Zr-based BMG with the composition of Zr_41.2_Ti_13.8_Cu_12.5_Ni_10_Be_22.5_ was remelted by electron beam welding. The microstructures of the heat affected zone (HAZ) and remelting zone (RZ) were analyzed. The thermal field of the electron beam welding was obtained by numerical simulation. The critical conditions for crystallization during the heating and cooling processes were obtained by differential scanning calorimetry (DSC) and the Kissinger equation. Finally, the crystallization mechanisms of both the heating and the cooling processes were discovered.

## 2. Materials and Methods

**Figure 1:**
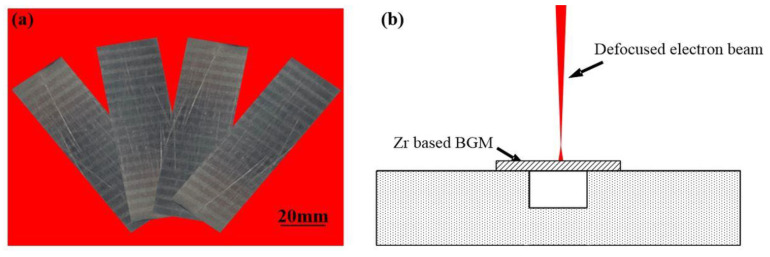
(**a**) The Zr_41.2_Ti_13.8_Cu_12.5_Ni_10_Be_22.5_ plate used in the trail and (**b**) the schematic diagram of the remelting process.

The quinary Zr_41.2_Ti_13.8_Cu_12.5_Ni_10_Be_22.5_ was produced by alloying the pure elements of Zr, Ti, Cu, Ni and Be in an induction furnace (EnigMa, Nanjing, China). The ingots were then remelted at least twice to ensure their chemical homogeneity. After remelting, the Zr_41.2_Ti_13.8_Cu_12.5_Ni_10_Be_22.5_ ingot was cut into small plates of size 50 × 20 × 1 mm as shown in Figure 1a, and then polished. The remelting process was conducted by a defocused electron beam welding machine (AVIC, Beijing, China) with a vacuum of 3 × 10^−3^ Pa as shown in Figure 1b. The defocused electron beam was chosen to eliminate the groove at the surface. The acceleration voltage was 60 kV. The welding beam current was 3 mA. The remelting speeds ranged from 11 to 13 mm/s, respectively. After welding, cross sections of the remelted pieces were cut and polished. Microstructures and compositions of the remelted zone were determined using an optical microscope (OM, Olympus—dsx510, Osaka, Japan) and scanning electron microscopy (SEM, Quanta 200FEG, FEI, Hillsboro, OR, USA) with an energy dispersive X-ray spectrometer (EDS, FEI, Hillsboro, OR, USA). The acceleration voltage was set as 20 kV, and the working distance was 10–12 mm. To test the phase transformation process of Zr-based BMGs, the Zr-based BMGs were cut into small blocks of the size 5 × 5 × 5 mm, and then tested by differential scanning calorimetry (DSC, Netzsch, Bavaria, Germany) with different heating and cooling rates.

**Figure 2:**
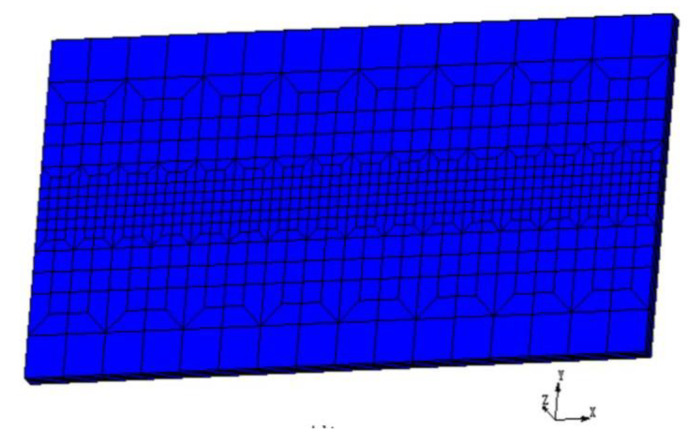
Finite element mesh generation of the welded plate.

The 3-D finite element model was employed to analyze the thermal fields of the electron beam remelting process. Non-uniform hexahedral mesh was adopted to build the model to balance the calculation efficiency and accuracy. Finite element mesh used in the work is shown in Figure 2. The heat transfer governing equation for the electron beam welding process is shown as following [15]

(1) $$\rho C\frac{\partial T}{\partial t}=\frac{\partial }{\partial x}(k\frac{\partial T}{\partial x})+\frac{\partial }{\partial y}(k\frac{\partial T}{\partial y})+\frac{\partial }{\partial z}(k\frac{\partial T}{\partial z})+\bar{Q}$$

where *x*, *y*, *z* are the Cartesian coordinates; *r* is the density; *c* is the specific heat; *k* is the thermal conductivity; *T* is the transient temperature; $\bar{Q}$ is the heat source intensity in the material. As the penetration ability of the defocused electron beam was limited, the double ellipsoidal heat source was used in this study

(2) $$q_{z}(x,y,z)=\frac{6\sqrt{3}f_{1}Q}{\pi a_{f}bc\sqrt{\pi }}\exp(-3\frac{x^{2}}{a_{f}^{2}}-3\frac{y^{2}}{w^{2}}-3\frac{z^{2}}{d^{2}})$$

(3) $$q_{z}(x,y,z)=\frac{6\sqrt{3}f_{2}Q}{\pi a_{r}bc\sqrt{\pi }}\exp(-3\frac{x^{2}}{a_{r}^{2}}-3\frac{y^{2}}{w^{2}}-3\frac{z^{2}}{d^{2}})$$

where *Q* is the heat input (*Q* = *UI*), *a_f_*, *a_r_*, *w*, and *d* are ellipsoidal heat source parameters, *f*_1_ and *f*_2_ are proportion coefficients representing heat apportionment in the front and the back of the heat source, respectively (*f*_1_ + *f*_2_ = 2). To guarantee the calculation’s accuracy, the following conditions were assumed according to Wang et al. [16]. (1) The materials are isotropic continuum materials. (2) The initial temperature is 25 °C. (3) Welding is a quasi-steady process. (4) The welding pool flow is omitted. (5) The heat transfer coefficient is 40 W/m^2^ K. Besides, the thermal properties of the Zr-based BMGs used for the calculations were similar to that of Yamasak et al [17].

## 3. Results

### Microstructures of the EB Remelted Zr-Based BMG

**Figure 3:**
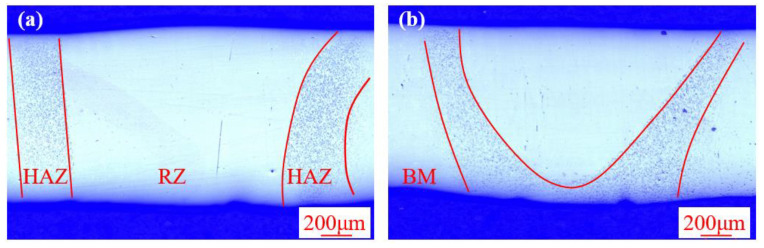
The cross-section morphology of the electron beam remelted Zr-based bulk metallic glasses (BMG) obtained by (**a**) 3 mA/11 mm·s^−1^ and (**b**) 3 mA/13 mm·s^−1^.

Figure 3 shows the cross-section morphology of the electron beam remelted Zr-based BMG obtained by different remelting velocities. Macroscopically, both the two sections display defect-free morphologies with a relatively smooth surface, indicating the effectiveness of the defocused electron beam in eliminating the groove defect. The remelting part, which consists of theheat-affected zone (HAZ) and remelting zone (RZ), can be clearly observed. The RZ shows a similar appearance with base metal (BM) while the HAZ appears unique morphology, with many small dots distributed on it. Moreover, the shape of the RZ is sensitive to the variation in the remelting velocity. When the remelted velocity was 11 mm·s^−1^, the RZ completely penetrated the plates, while the RZ partially penetrated the plate when the remelted velocity increased to 13 mm·s^−1^.

**Figure 4:**
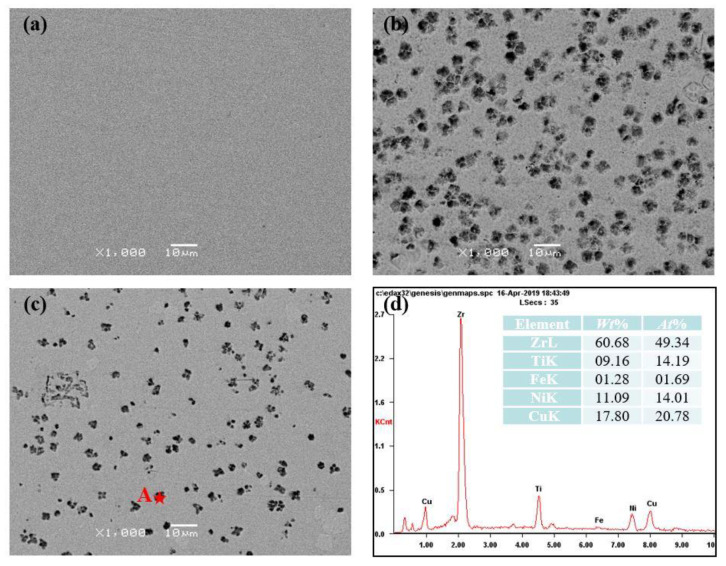
The different morphologies of (**a**) the remelting zone (RZ), (**b**) the heat-affected zone (HAZ) near the RZ and (**c**) away from the RZ. (**d**) shows the EDS results of point “A”.

To further analyze the difference between the HAZ and the RZ, the SEM was used to observe the microstructures. Figure 4 shows the different morphologies of the RZ and the HAZ, near and away from the RZ. No contrast was observed in Figure 4a, indicating the amorphous feature of the RZ. Some small dots, several microns in size, formed in the HAZ as shown in Figure 4b,c, which demonstrates the crystallization of Zr_41.2_Ti_13.8_Cu_12.5_Ni_10_Be_22.5_. Further comparing the number of dots in the HAZ shows the severe crystallization in the zone near the RZ. The EDS results (Figure 4d) of the small dots in the HAZ prove that the molar ratio of Zr + Ti and Cu + Ni is approximately 2:1, indicating the formation of (Ti, Zr)_2_(Cu, Ni). This is consistent with previous results of Schroers et al. [18]. The different crystallization degrees in the HAZ and the RZ may be attributed to the different thermal cycle process during the remelting process. Meanwhile, it is still unclear whether the crystallization in the HAZ occurs during the heating process or the cooling process.

## 4. Discussion

**Figure 5:**
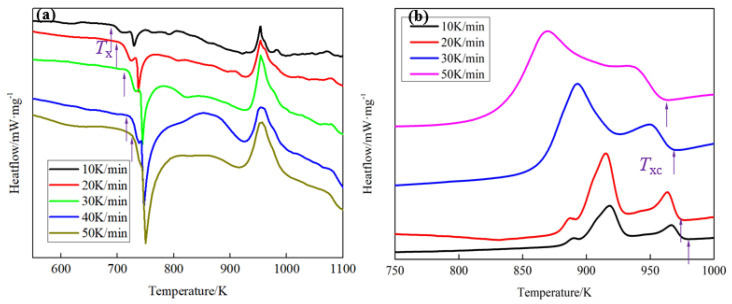
The differential scanning caliometry (DSC) curves with different (**a**) heating rate and (**b**) cooling rate.

**Figure 6:**
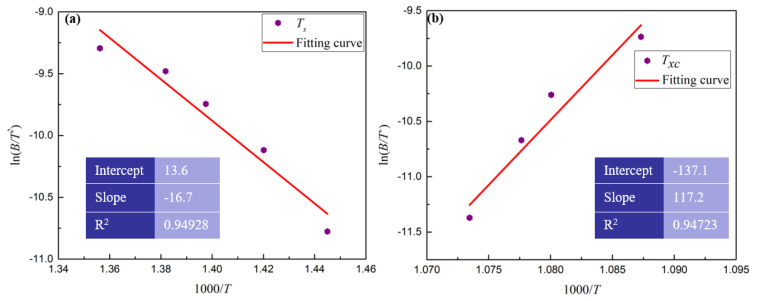
The linear fit between ln(*B*/*T*^2^) and 1000/*T* during (**a**) heating and (**b**) cooling process.

The research of Li et al. shows that the crystallization of BMGs is determined by glass forming ability [19]. To analyze the glass forming ability of Zr_41.2_Ti_13.8_Cu_12.5_Ni_10_Be_22.5_, the Kissinger equation was used to calculate its activation energy, as the Zr-based bulk metallic glasses often crystallizes by simultaneous precipitation of Zr_2_Ni, Zr_2_Cu, Cu_10_Zr_7_ and other crystalline phases, which is a complicated polymorphic crystallization process. The empirical formula (Equation (4)) to calculate activation energy which is put forward by Kissinger, is shown as the following [20]

(4) $$\ln\left(\frac{B}{T^{2}}\right)=-\frac{E}{RT}+C$$

where *B* is the heating or cooling rate; *T* is the specific temperature; *R* is the gas constant; and *E* is the activation energy. It is obvious that there is a linear relationship between ln(*B*/*T*^2^) and 1/(*RT*). The activation energy (*E*) is the slope. The specific temperature, such as the crystallization temperature (*T*_x_, *T*_xc_) during the heating or cooling process, was tested by DSC and the results are shown in Figure 5 and Table 1. The linear regression model was used to estimate the activation energy (*E*) and the results are shown in Figure 6. As is shown in Figure 6, the absolute value of the slope of the linear relationship between ln(*B*/*T*^2^) and 1000/*T* in the heating process is 16.7, while that of the cooling process is 117.2. Thus, the calculated activation energy (*E*) in the heating and cooling process is 139.19 and 975.02 kJ/mol, respectively. Thus, the BMGs are more difficult to crystallize at *T*_xc_ than that at *T*_x_. Namely, it is difficult to crystallize by liquid–solid phase transformation at *T*_xc_, which is consistent with the results in Figure 3 and Figure 4.

The research on activation energy shows that the BMG in the HAZ is prone to crystallization. However, the HAZ underwent both the heating and the cooling processes during the remelting process. It is necessary to figure out whether the BMG in the HAZ crystallize in the heating process or in the cooling process. Thus, the critical heating rate and cooling rate of crystallization by solid–solid phase transformation are calculated. Taking the heating process as an example to calculate the critical heating rate, the average heating rate (*B*) can be described as the following

(5) $$B=\left(\frac{T-293}{t}\right)$$

where *T* is the specific temperature and *t* is the heating time. The crystallization characteristic temperature for BMGs during the heating process is *T_x_*. As the heating rates were set as 10, 20, 30, 40 and 50 K/min, it took 2400, 1236, 848, 648 and 533 s, respectively, heating the BMGs from room temperature to *T_x_* during the DSC test as shown in Table 2.

**Figure 7:**
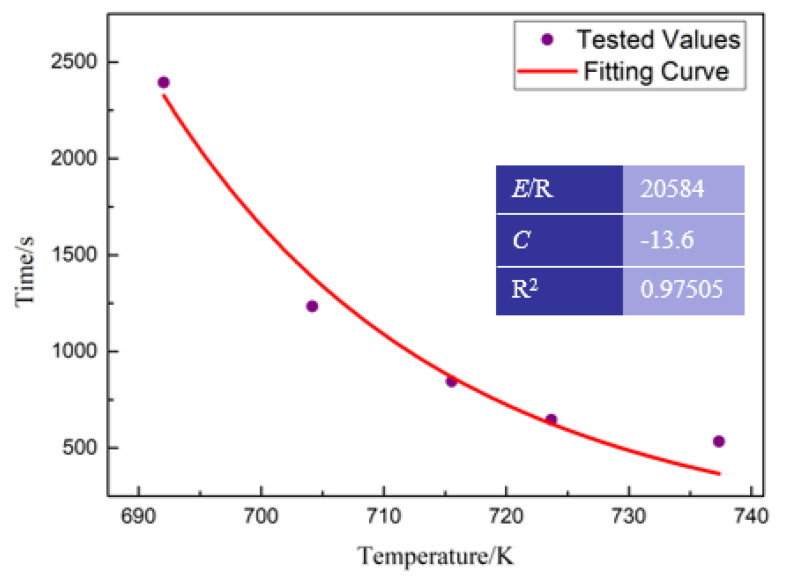
The fitting curve between *T*_x_ and *t.*

Substituting Equation (5) into Equation (4), the relationship between specific temperature and heating time can be written as

(6) $$\ln\frac{T^{2}t}{T-293}=\frac{E}{RT}+C$$

where the values of *E* and *C* can be obtained by fitting the relationship between *T_x_* and *t* in Table 2. The results are shown in Figure 7. Thus, the mathematical expression for describing the relationship between *T_x_* and *t* can be determined as the following

(7) $$\ln\frac{T^{2}t}{T-293}=\frac{20584}{T}-13.6$$

If the Zr_41.2_Ti_13.8_Cu_12.5_Ni_10_Be_22.5_ did not crystallize during the remelting process, the melting point was the only specific temperature during the DSC test. Thus, the critical heating time between room temperature and melting point was calculated. The tested melting point (about 931 K) of Zr_41.2_Ti_13.8_Cu_12.5_Ni_10_Be_22.5_ during heating process was obtained by DSC. The critical heating time, which was calculated by Equation (5), was 2.98 s. Therefore, the critical rate, which was calculated by Equation (3), to avoid the crystallization of Zr_41.2_Ti_13.8_Cu_12.5_Ni_10_Be_22.5_ by solid–solid phase transformation was 218 K/s.

**Figure 8:**
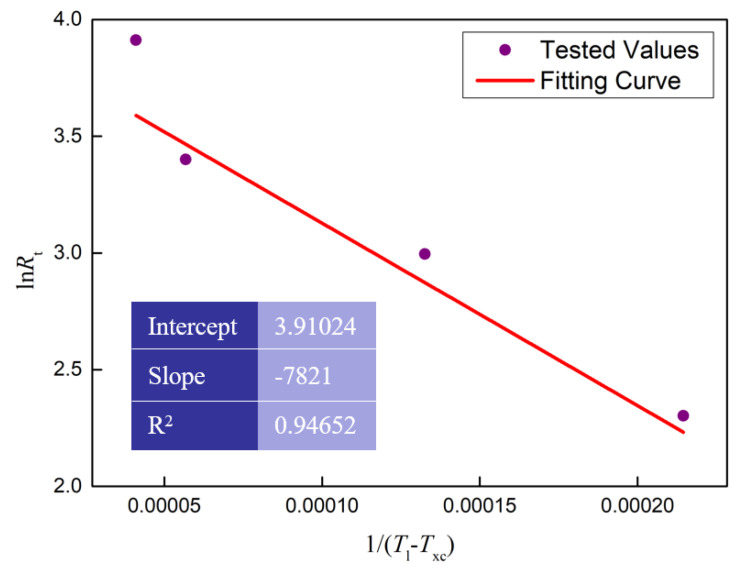
The linear fitting curve between ln*R_t_* and 1/(*T_l_* − *T_xc_*)^2^ during cooling process.

The research of Barndiaran et al. showed that the critical cooling rate to avoid the crystallization of Zr_41.2_Ti_13.8_Cu_12.5_Ni_10_Be_22.5_ by liquid–solid phase transition can be calculated by the following equation [21]

(8) $$\ln R_{t}=\ln R_{c}-\frac{b}{{\left(T_{1}-T_{xc}\right)}^{2}}$$

where *R_t_* is the cooling rate (K/s); *R_c_* is the critical cooling rate (K/s); *T_l_* is the end temperature of the melting process (K); *T_xc_* is the onset temperature of the solidification process (K); the parameter *b* is constant. It is obvious that there is a linear relationship between ln*R_t_* and 1/(*T_l_* − *T_xc_*)^2^. The values of *R_t_*, *T_l_* and *T_xc_* were obtained by the DSC test as shown in Figure 5b. The intercept of the linear relationship between ln*R_t_* and 1/(*T_l_* − *T_xc_*)^2^ reflects the critical cooling rate. The linear regression model was used to estimate the critical cooling rate, and the results are shown in Figure 8. According to the liner fitting results in Figure 8, the critical cooling rate was calculated as 0.98 K/s.

**Figure 9:**
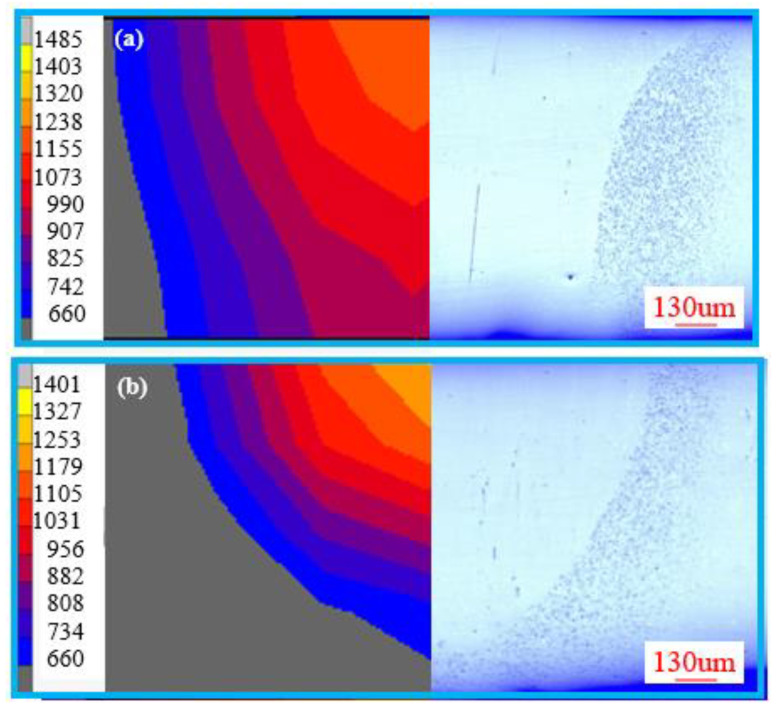
The typical thermal fields’ morphologies and cross section of remelted BMGs obtained by the remelting rate of (**a**) 11 mm/s and (**b**) 13 mm/s.

The theoretical critical heating or cooling rates for the crystallization of Zr_41.2_Ti_13.8_Cu_12.5_Ni_10_Be_22.5_ were obtained above. The real time heating and cooling rates during the actual remelting process determined whether the crystallization occurred or not. Thus, the 3-D finite element model was employed to analyze the real time heating and cooling rates of the electron beam welding process. Figure 9 compares the typical thermal fields’ morphologies and the cross-section of remelted BMGs. It is obvious that the width at the front and back side of the simulated results are similar to that of the experiments, which verifies the accuracy of the FEM.

**Figure 10:**
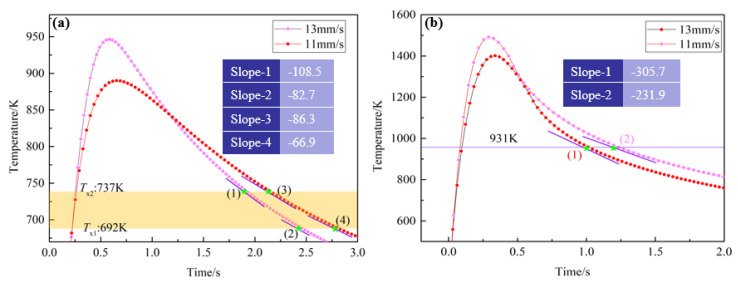
The typical thermal curves of the points located in (**a**) HAZ and (**b**) RZ.

Figure 10 shows the thermal cycle curves of the electron beam remelting process obtained by different remelting speeds. It is obvious in Figure 10a that the heating rates at *T*_x_ in HAZ are faster than the critical heating rate (218 K/s), as it only takes 0.58 and 0.65 s to heat the HAZ from room temperature to peak temperature during the electron beam remelting process, with a remelting rate of 13 and 11 mm/s, respectively. Thus, the Zr-based BMGs did not undergo the solid–solid phase transformation during the heating process. Namely, crystallization of Zr-based BMGs did not occur in the heating process. However, the maximum cooling rate of the HAZ obtained by 13 and 11 mm/s is 204.7 and 121.7 K/s, respectively. The cooling rate is lower than the critical cooling rate. Thus, the Zr-based BMGs in the HAZ have crystallized during the cooling process. Similarly, for the Zr-based BMGs in the RZ, the cooling rate around the melting point is obviously faster than the calculated critical cooling rate (0.98 K/s), as shown in Figure 10b. Thus, the Zr-based BMGs in the RZ keep their amorphous state after the remelting process.

## 5. Conclusions

The Zr_41.2_Ti_13.8_Cu_12.5_Ni_10_Be_22.5_ underwent severe crystallization in the HAZ and the RZ during the electron beam remelting process. The critical conditions for crystallization during the heating and cooling processes were obtained by DSC and Kissinger equation. The crystallization mechanisms of both the heating and the cooling process were discovered by comparing the critical conditions and calculated results. This work provides the fundamental physical method for understanding the relationship between process parameters and the crystallization of BMGs, which might offer solutions to the size limitation in the application of BMGs.

(1) The Zr_41.2_Ti_13.8_Cu_12.5_Ni_10_Be_22.5_ in the HAZ undergoes severe crystallization, while the Zr_41.2_Ti_13.8_Cu_12.5_Ni_10_Be_22.5_ in the RZ keeps its amorphous state after the remelting process. The crystallized phase was verified as (Ti, Zr)_2_(Cu, Ni);
(2) The critical heating or cooling rate of Zr_41.2_Ti_13.8_Cu_12.5_Ni_10_Be_22.5_ to avoid crystallization by solid–solid phase transformation was 218 K/s. The critical cooling rate to avoid crystallization by liquid-solid phase transition was calculated as 1.2 K/s;
(3) The heating rates at Tx in the HAZ are faster than the critical heating rate (218 K/s), while the cooling rates at Tx in the HAZ are lower than critical cooling rate. The HAZ crystallized during the cooling process. The cooling rate around the melting point is faster than the calculated critical cooling rate (1.2 K/s). The Zr-based BMGs in the RZ keep their amorphous state after the remelting process.

## Figures and Tables

**Table 1:** The crystallization temperature during the heating and cooling process.

Heating or Cooling Rate (*B/*(K/min))	Specific Temperature	
	Crystallization Temperature in during Heating Process (*T_x_*/K)	Crystallization Temperature in during Cooling Process (*T_xc_*/K)
10	692.05	931.57
20	704.16	927.95
30	715.54	925.88
40	723.68	/
50	737.35	919.70

**Table 2:** The crystallization temperature and heating time of Zr-based BMGs at different heating rates.

Heating Rate (*B/*(K/min))	Crystallization Temperature in during Heating Process (*T_x_*/K)	Heating Time (*t*/s)
10	692.05	2400
20	704.16	1236
30	715.54	848
40	723.68	648
50	737.35	533
